# An Evaluation of the Mechanisms of Galacto-Oligosaccharide (GOS)-Induced IgE Cross-Linking on Basophils in GOS Allergy

**DOI:** 10.3389/falgy.2022.840454

**Published:** 2022-02-28

**Authors:** Li Yuan Gabriella Nadine Lee, Si Yuan Leow, Hongmei Wen, Jian Yi Soh, Wen Chin Chiang, Youjia Zhong, Elizabeth Huiwen Tham, Wenyin Loh, Dianne J. Delsing, Bee Wah Lee, Chiung-Hui Huang

**Affiliations:** ^1^Department of Paediatrics, Yong Loo Lin School of Medicine, National University of Singapore, Singapore, Singapore; ^2^Khoo Teck Puat-National University Children's Medical Institute, National University Health System, Singapore, Singapore; ^3^Department of Paediatrics, Kandang Kerbau Women's and Children's Hospital, Singapore, Singapore; ^4^FrieslandCampina, Amersfoort, Netherlands

**Keywords:** galacto-oligosaccharides, galacto-oligosaccharides allergy, IgE cross-linking, basophils, galectin inhibitors

## Abstract

The prebiotics, galacto-oligosaccharides (GOS), are small carbohydrate molecules with 1–7 galactose units linked to glucose and have been shown to trigger IgE-mediated anaphylaxis in some cases following ingestion. It is still an unresolved question of how GOS cross-links IgE on basophils. In this study, we examined whether human galectins, a class of lectins that bind specifically to β-galactoside carbohydrates, are involved in GOS-induced basophil activation. Basophil activation test to GOS and control allergen, *Blomia tropicalis* (Blo t) extract were performed in the presence or absence of four sugar-based galectin inhibitors (lactose, thiodigalactoside [TDG], TD139, and GB1107) and one peptide-based inhibitor, G3-C12. Results showed that TD139, GB1107, and G3-C12 did not display a specific inhibitory effect on GOS-induced basophil activation as compared to control allergen. An inhibitory effect of lactose and TDG on GOS-induced basophil activation was observed and varied between subjects with up to 100% inhibition at low doses of GOS. The results of competitive ELISA suggest that the inhibitory effects of high dose lactose and TDG on the basophil activation is likely due to the cross-reactivity of GOS-specific IgE to lactose and TDG. Basophil activation is performed using purified basophils suggested that cell surface receptors on other blood cells were not required to induce basophil activation. In conclusion, our results suggest that GOS, a low molecular weight sugar, is able to cross-link IgE independently.

## Introduction

Galacto-oligosaccharides (GOS) are prebiotics, which are used widely supplemented in commercial infant formula and beverages for their ability to selectively promote the growth of beneficial gut bacteria in the human intestine, such as bifidobacteria (1). GOS is a complex mixture consisting of oligosaccharides with 1 up to 7 galactose units linked to a glucose terminal. The degree of polymerization (DP) of an oligosaccharide reflects the number of sugar units (2). GOS synthesized commercially from β-galactosidase enzyme derived from *Bacillus circulans* consists mainly of β-1,4-linked GOS. Other β-glycosidic linkages between the monomer units such as 1-6 Gal, 1-2 Glc, 1-3 Glc, 1-4 Glc, 1-6 Glc, 1-2 Gal, and 1-3 Gal also exist and can form linear and branched linkages (3). The structures and graphical representation of the permutations of linkages for DP2, DP3, and DP4 are depicted in Table 1.

**Table 1:** Structures and graphical representation of disaccharides, trisaccharides, and tetrasaccharides present in the Vivinal^®^ galacto-oligosaccharide (GOS) DP2, DP3, and DP4 pools (3).

**Disaccharides (DP2)**		**Trisaccharides (DP3)**		**Tetrasaccharide (DP4)**	
**Structures**	**Graphical presentation**	**Structures**	**Graphical presentation**	**Structures**	**Graphical presentation**
Galβ1-2Glc	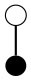	Galβ1-4Galβ1-2Glc	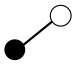	Galβ1-4Galβ1-4Galβ1-2Glc	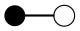
Galβ1-3Glc	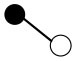	Galβ1-4Galβ1-3Glc	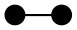	**Galβ1-4Galβ1-4Galβ1-3Glc**	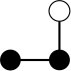
Galβ1-4Glc	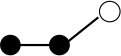	Galβ1-4Galβ1-4Glc	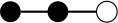	Galβ1-4Galβ1-4Galβ1-4Glc	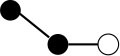
		Galβ1-6Galβ1-4Glc	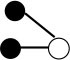	Galβ1-4Galβ1-6Galβ1-4Glc	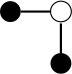
		Galβ1-4(Galβ1-6)Glc	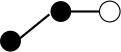	Galβ1-4Galβ1-4(Galβ1-6)Glc	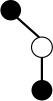
				**Galβ1-4Galβ1-6(Galβ1-4)Glc**	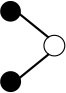
		Galβ1-2(Galβ1-4)Glc	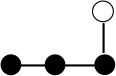	Galβ1-4Galβ1-2(Galβ1-4)Glc	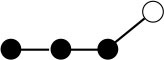
				Galβ1-4Galβ1-4(Galβ1-2)Glc	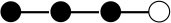
		Galβ1-3Galβ1-4Glc	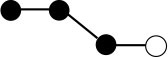		
Galβ1-6Glc	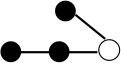	Galβ1-2(Galβ1-6)Glc	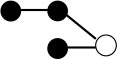	Galβ1-4Galβ1-2(Galβ1-6)Glc	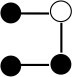
				Galβ1-4Galβ1-6(Galβ1-2)Glc	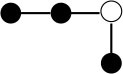
		Galβ1-3(Galβ1-6)Glc	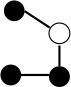		
				Galβ1-4Galβ1-4Galβ1-6Glc	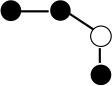
Galβ1-4Gal	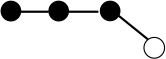				

*° indicates glucose (Glc); • indicates galactose (Gal)*.

*The allergenic GOS structures for GOS allergy patients in Japan were shown in bold*.

Soon after the introduction of GOS supplemented infant and maternal milk formula to South East Asia, cases of IgE-mediated immediate allergic reactions and anaphylaxis linked to GOS were reported (4, 5). These reactions occurred upon first consumption of these products and the putative primary sensitizer has been determined to be *Blomia tropicalis* (Blo t) (6).

GOS is a unique allergen as it is a pure carbohydrate. The conventional allergen is a protein or glycoprotein that is capable to initiate T cell responses and bind allergen specific IgE. Even though a vast diversity of anti-glycan IgE is detectable in circulation, the binding of this IgE to cross-reactive carbohydrate determinants have been shown to be weak in affinity and, therefore, clinically irrelevant (7). This concept was challenged with the description of allergy to alpha-gal, where IgE to this carbohydrate epitope resulted in Cetuximab-induced anaphylaxis (8).

In contrast to α-gal, an epitope that lies within a glycoprotein through N-glycosylation (9); GOS is a more unusual allergen as it is a pure carbohydrate with an average molecular weight of <1,000 daltons. For effective activation of basophils, allergens require at least two IgE epitopes for cross-linking of IgE (10). It is still an unresolved question how a small carbohydrate like GOS cross-links IgE. This study aimed to evaluate the possible mechanisms by which GOS activates basophils; specifically, we determined if cell surface proteins on basophils and/or other immune cells act as carrier proteins to cross-link IgE. Galectins on cell surfaces were selected as a plausible candidate, as they are a class of lectins that bind specifically to β-galactoside carbohydrates *via* a carbohydrate recognition domain (11). In this study, we show that cross-linking of IgE on basophils by GOS does not require the binding of GOS to galectins or to other cell surface proteins. We speculate that GOS itself is, therefore, likely to be able to cross-link IgE on basophils.

## Materials and Methods

### Reagents

Galectin inhibitors used in this study were β-lactose (Sigma Aldrich, St. Louis, Mo), thiodigalactoside (TDG) (Cayman Chemical, Ann Arbor, MI), TD-139 (Selleckchem, Houston, TX), GB1107 & G3-C12 (MedChemExpress, Monmouth Junction, NJ). Their structures and binding affinities to galectins were shown in Table 2. All are sugar-based inhibitors, except G3-C12 which is a peptide. Lactose binds to most structures of galectins with low affinity (dissociation constant [*K*d] at micromolar to millimolar range). TDG binds to galectin-1,−2,−3,−8,−9 with slightly higher affinity than lactose (*K*d at micromolar range). Inhibitors TD139 and GB1107 have similar targets as TDG but have a higher affinity to galectin-3 (*K*d at nanomolar range) selectively. G3-C12 binds to galectin-3 specifically at high affinity. Anti-human monoclonal antibodies used for BAT assay were purified α-IgE (G7-18, BD Biosciences, Franklin Lakes, NJ), fluorescein isothiocyanate (FITC) labeled α-CD63 (H5C6; BioLegend, San Diego, CA), allophycocyanin (APC) labeled α-CD203c (NP4D6; BioLegend), phycoerythrin (PE) labeled α-IgE PE (Ige21; eBioscience, Carlsbad, CA). Vivinal^®^ GOS syrups were obtained from FrieslandCampina, The Netherlands. A fraction of GOS with enriched allergenic GOS DP4 structures and Blo t extract were prepared as previously described (6).

**Table 2:** Panel of galectin (Gal) inhibitors used in this study.

**Name**	**Structure**	Targets^*^	***K_***d***_* (μM)**	**References**
Lactose	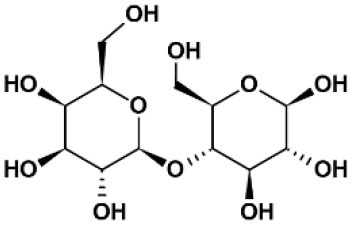	Gal-1	330	(12)
		Gal-2	85	(13)
		Gal-3	26-1000	(13)
		Gal-8	150	(14)
		Gal-9	N.A.	(15)
		Gal-12	N.A.	(16)
TDG	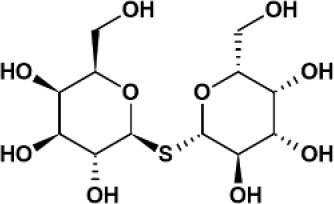	Gal-1	24	(17)
		Gal-2	340	
		Gal-3	49	
		Gal-8	61	
		Gal-9	38	
TD 139	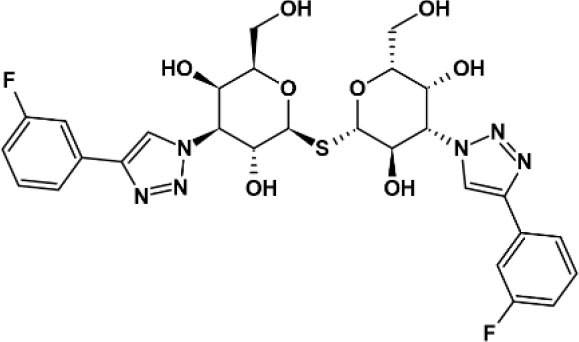	Gal-1	0.012	(17)
		Gal-2	>5	
		Gal-3	0.014	
		Gal-8	86	
		Gal-9	0.68	
GB 1107	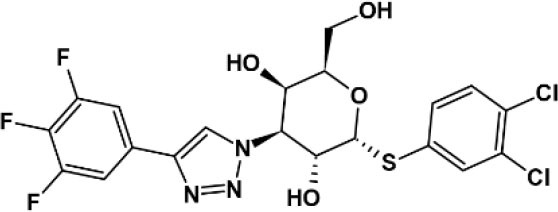	Gal-1	3.7	(18)
		Gal-2	0.64	
		Gal-3	0.037	
		Gal-8	83	
		Gal-9	2.4	
G3-C12	Ala-Asn-Thr-Pro-Cys-Gly-Pro-Tyr-	Gal-3	0.088	(19)
	Thr-His-Asp-Cys-Pro-Val-Lys-Arg			

*, *Targets involved galectins expressed by immune cells*. *N.A., not available*.

### GOS Allergic Subjects

A total of nine subjects allergic to GOS were recruited in this study. They are either patients who developed allergic symptoms after consuming GOS containing milk (*n* = 5) or subjects who developed allergic symptoms upon GOS oral challenge (*n* = 4). The clinical features of these subjects were shown in Table 3.

**Table 3:** Clinical features of GOS sensitized subjects.

**No**	**Age of onset (years)**	**Dose eliciting reaction (gram)**	**Symptoms**	**Time to onset of symptom (minutes)**	**SPT (mm)**		
					**vGOS**	**Blo t**	**Der p**
S1^#^	26	0.7	AE, RS, Resp	40	5 × 5	5 × 4	5 × 5
S2^#^	13	1.5	AE, LU, GI, Respi	10	10 × 8	10 × 5	15 × 15
S3^#^	9	0.96	AE	5	5 × 6	4 × 4	4 × 4
S4^*^	27	0.6	AE, RS, Respi	30	8 × 5	6 × 6	5 × 9
S5^#^	7	0.64	AE	30	5 × 5	5 × 5	5 × 5
S6^*^	30	2	AE, GU, RS	30	4 × 5	5 × 5	5 × 5
S7^#^	38	1.5	AE, RS, Resp	10	5 × 4	5 × 6	5 × 6
S8^*^	40	2	AE, RS	30	5 × 6	5 × 4	5 × 5
S9^*^	43	2	Respi	30	5 × 8	5 × 5	5 × 4

*LU, localized urticaria; AE, angioedema; GU, generalized urticaria; RS, rhinorrhoea and bouts of sneezing; Resp, wheeze, dry persistent cough, or dyspnoea; GI, vomiting*.

# *GOS allergic patients with historical clinical reactions*.

* *Subjects have allergic symptoms upon vGOS oral challenge*.

### Basophil Activation Assay With Galectin Inhibitors

**Figure 1:**
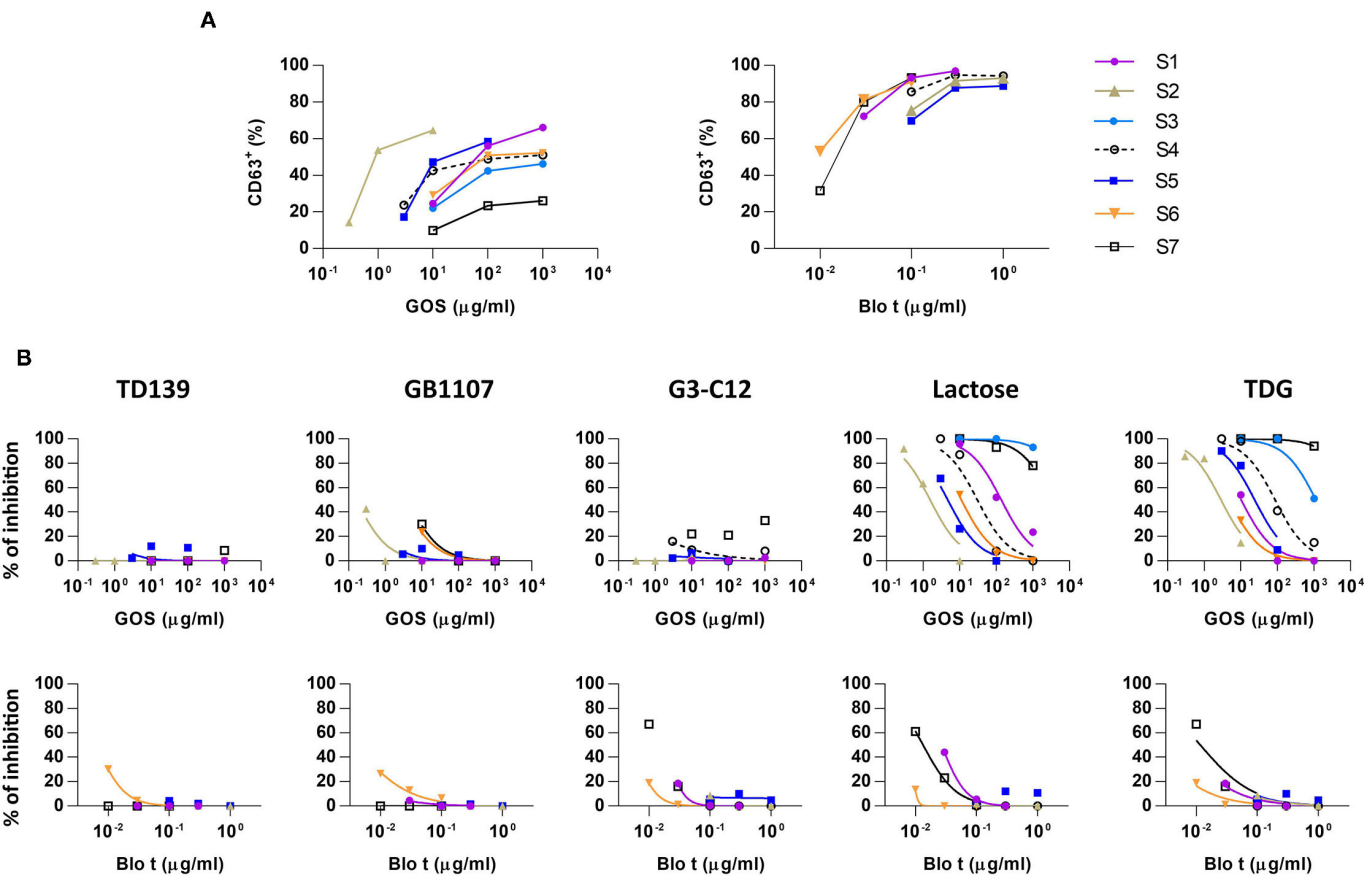
Basophil activation assays to galacto-oligosaccharide (GOS) and *Blomia tropicalis* (Blo t) in the absence or presence of galectin inhibitors. **(A)** Dose response curve for the expression of CD63 upon GOS or Blo t stimulation. Heparinized whole blood was stimulated with various concentration of GOS (*n* = 7) or Blo t (*n* = 6). The expression of CD63 on basophils was analyzed by flow cytometry. **(B)** Dose response curve for the expression of CD63 upon GOS or Blo t stimulation in the presence of 50 μM of TD139 (*n* = 5), 50 μM of GB1107 (*n* = 5), 50 μM G3-C12 (*n* = 6), 100 mM of lactose (*n* = 7 for GOS; *n* = 6 for Blo t), or 50 mM TDG (*n* = 7 for GOS; *n* = 6 for Blo t).

Basophil activation assay was performed using sodium heparinized blood from subjects who were allergic to GOS. The galectin inhibitors, basophil stimulants (GOS, Blo t extract and anti-IgE) were prepared at four times concentrated solution (to the final concentration shown in Figure 1). The doses of inhibitors used in this study were at least 500 times higher than their *K*d value to galectin-3, except for GB1107 and lactose. A concentration of 250 times higher than *K*d for galectin-3 was used due to its relatively poor solubility in aqueous solution. For lactose, a concentration of 100 mM was used as this concentration was shown to inhibit the binding of galectins to cell surface of Chinese hamster ovary (CHO) cells (20). Fifty microliters of concentrated galectin inhibitors or phosphate-buffered saline (PBS) were added into 100 μl of whole blood aliquots. The samples were pre-incubated at 37°C for 10 min. Thereafter, 50 μl of concentrated vGOS, Blo t extract, PBS (negative control) or anti-IgE (positive control) were added into the inhibitor-blood mixture. Samples were then incubated at 37°C for additional 15 min. The reaction was stopped by adding 1 ml of 1% BSA/PBS-EDTA (20 mM) to each tube. After washing the cells with 1% bovine serum albumin (BSA) (Sigma Aldrich)/PBS, cells were stained with FITC labeled anti-CD63, PE labeled anti-IgE, and APC labeled anti-CD203c mAbs for 20 min at 4°C. Thereafter, samples were subjected to erythrocyte lysis with 2 ml of FACS lysing solution (Becton Dickinson, San Jose, CA, USA). Cells were then washed, resuspended in 1% BSA/PBS and analyzed by FACScan (Becton Dickinson). Basophils were identified as IgE^high^ and CD203c^+^ cells. The percentage of inhibition was calculated as (1 – ΔCD63${}_{\left(\text{with inhibitor}\right)}^{+}$/ΔCD63${}_{\left(\text{without inhibitor}\right)}^{+}$) × 100. ΔCD63^+^ = (% of CD63^+^ with stimulant – % of CD63^+^ without stimulant).

### Basophil Activation Assay Using Purified Basophils

Peripheral blood mononuclear cells (PBMCs) were isolated from heparinized blood by Ficol-Paque density gradient centrifugation. Basophils were then isolated from PBMCs using basophil isolation kit II and LS column according to manufacturer's instruction (Miltenyi Biotec, Germany). The purity of basophils was more than >80% as analyzed by flow cytometry. Purified basophils were suspended in 1% BSA/Dulbecco's DPB (D-PBS) in a concentration of 1 × 10^5^ cells/ml supplemented with 4 ng/ml of IL-3 (Miltenyi Biotec). To perform the BAT assay, 100 μl of purified basophils aliquots were pre-incubated at 37°C for 10 min. Thereafter, 100 μl of vGOS, Blo t extract, PBS (negative control), or anti-IgE (positive control) were added into the tube. Samples were incubated at 37°C for 15 min, stained, and analyzed for the expression CD63^+^ using protocols as described in the previous section.

### Competitive IgE ELISA

Sera were incubated overnight with lactose or TDG at final concentrations ranging from 0.03 mM to 100 mM at 4°C. Self-inhibition of GOS-specific IgE was performed using Vivinal^®^ GOS syrup or the enriched tetrasaccharide GOS fraction (DP4) at 1 or 10 μg/ml (final concentration). The pre-absorbed sera were then tested for the level of GOS-specific IgE by ELISA developed in-house as previously described (6). The percentage of inhibition was calculated as (1-IgE level with inhibitor/IgE level without inhibitor) × 100.

## Results

### Evaluation of the Role of Galectin in GOS-Mediated Basophil Activation

Basophil activation tests to GOS and Blo t using whole blood from seven patients with GOS allergy and concomitant IgE sensitization to Blo t showed typical dose-dependent expression of CD63 (Figure 1A). To test whether cell surface galectins are involved in GOS-induced basophil activation as a carrier to cross-link IgE on basophils, a panel of five galectin inhibitors (Table 2) was used in the BAT assay. Whole blood samples were pre-incubated with inhibitors before adding GOS or Blo t. Three of the five inhibitors (TD139, GB1107, and G3-C12) showed minimal inhibitory effects for both GOS- and Blo t-mediated basophil activation (Figure 1B), indicating that basophil activation is not likely to involve galectins. In contrast, the two remaining galectin inhibitors, lactose and TDG, could inhibit GOS-induced basophil CD63 expression (Figure 1B). The inhibitory effects of lactose and TDG on GOS-induced basophil activation varied between subjects (ranging from 0 to 100%) and decreased with increasing concentrations of GOS. Inhibition by lactose and TDG on Blo t-mediated basophil activation was observed in 2/7 subjects at low doses (<0.1 μg/ml) of Blo t. As lactose and TDG have lower affinity for galectins compared to the rest in the panel, we speculated that their inhibitory effect on GOS-mediated basophil activation arose from a different mechanism, so the chemical structures of these 2 galectin inhibitors were then examined.

### Lactose and TDG Blocked the Binding of GOS to GOS-Specific IgE

**Figure 2:**
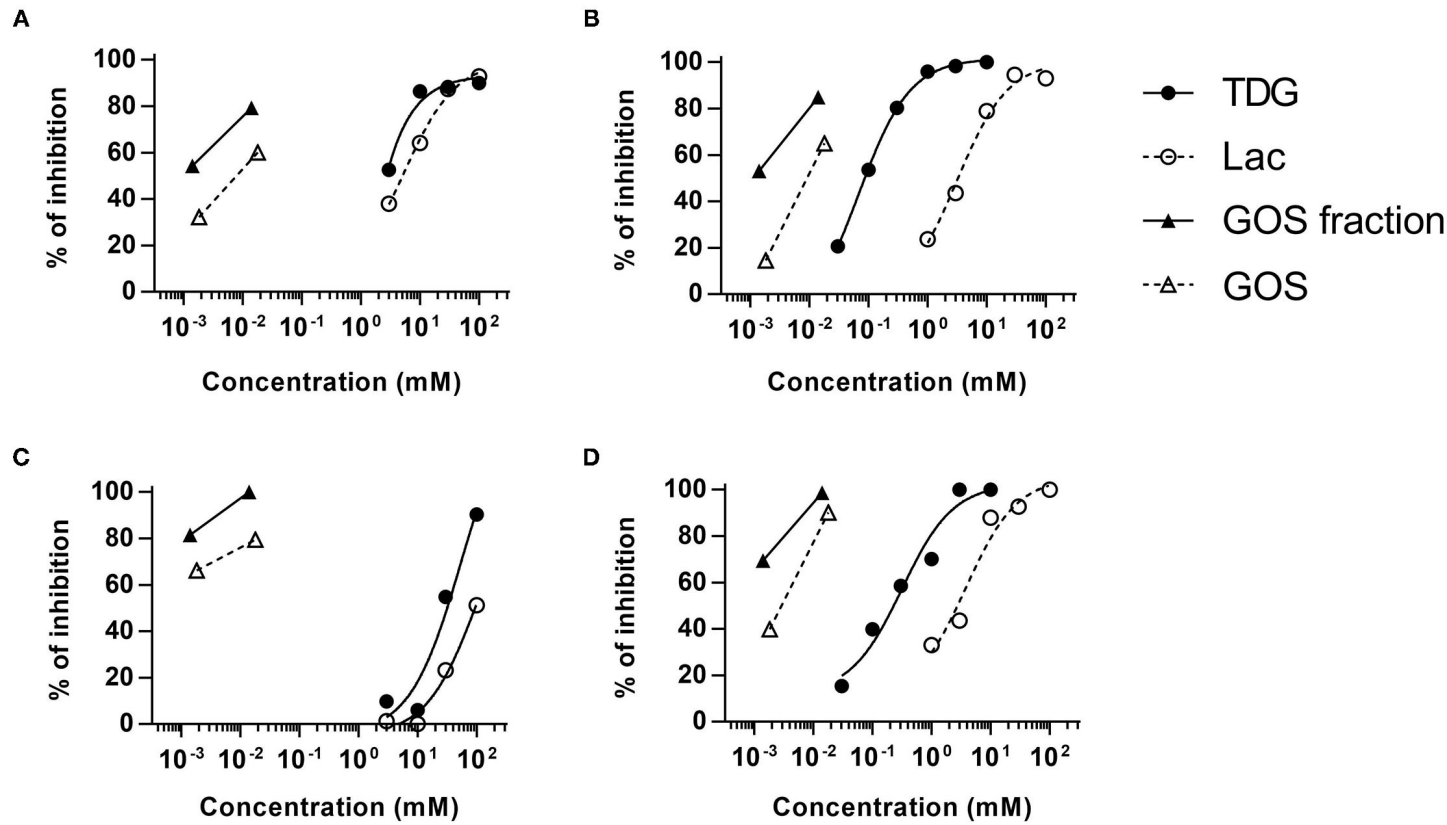
Competitive ELISA assay for GOS-specific IgE. Sera (*n* = 4) were pre-incubated with various doses of lactose, TDG, GOS, or allergenic GOS tetrasaccharides (GOS fraction) before the detection of GOS-specific IgE by ELISA. Graph **(A–D)** represent subjects S7, S8, S4, and S9, respectively.

Both lactose and TDG structures (Table 2) are disaccharides, consisting of galactose and β-linked glucose, and β-linked digalactoside, respectively. As these structures closely resemble GOS, we went on to evaluate if lactose and TDG could bind GOS-specific IgE using IgE ELISA inhibition assay. Patients' sera were pre-incubated with lactose or TDG before the ELISA detection of GOS-specific IgE. Self-inhibition for GOS-specific IgE was conducted using commercial GOS or using a GOS fraction-enriched in allergenicity as described earlier (6). This GOS fraction was the material used as coating antigen in the ELISA assay. Hence, it is a self-inhibition control. The results showed that both lactose and TDG inhibited the GOS-specific IgE. The concentration of lactose and TDG required to achieve 50% inhibition was between 5–100 mM and 0.1–55 mM, respectively (Figure 2). In contrast, concentrations as low as 0.01 mM and 0.002 mM for commercial GOS and enriched GOS fraction, respectively, could achieve 50% inhibition of GOS-specific IgE. The results indicate that TDG has higher affinity for GOS-specific IgE compared to lactose, and both these inhibitors have lower affinity than GOS itself. Hence, the observed inhibition of BAT to GOS by lactose and TDG at low doses of GOS (Figure 1B) is likely due to competitive binding to GOS-specific IgE.

### GOS Induces IgE Cross-Linking on Purified Basophils

**Figure 3:**
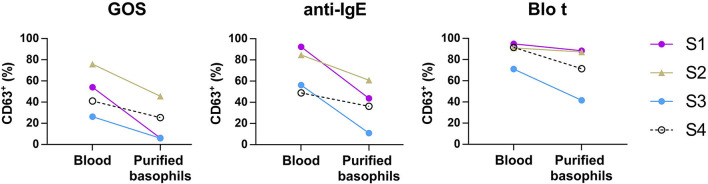
Basophil activation assay performed using purified basophils. Whole blood or purified basophils (*n* = 4) were stimulated with 100 μg/ml of GOS (**left**), 1 μg/ml of anti-IgE (**middle**), or 0.3 μg/ml of Blo t (**right**). The expression of CD63^+^ on basophils was analyzed by flow cytometry.

To test whether IgE cross-linking by GOS on basophils required other cell types to act as adjuvants, purified basophils were used in the BAT assay. Purified basophils were stimulated with GOS and two control stimulants (anti-IgE and Blo t). As compared to the BAT performed using whole blood, purified basophils were less sensitive to GOS stimulation and a higher concentration of GOS was required to induce basophil activation (Supplementary Figure 1). When basophils were stimulated with 100 μg/ml of GOS, there was 40–90% reduction in the percentage of CD63^+^ cells upon GOS stimulation (Figure 3). Two subjects still showed positive responses (>10% of CD63^+^ cells) to GOS and other two subjects showed almost negative BAT responses (<10%) in the assay using purified basophils. Similarly, there was 26–81% reduction in the percentage of CD63^+^ cells upon anti-IgE stimulation, with one subject having almost negative BAT responses (<10%). There was also a reduction (4–40%), albeit to a lesser degree, of CD63^+^ cells when cells were stimulated with a potent and multivalent allergen, Blo t. These results suggest that GOS may directly act on basophils and induce IgE cross-linking without the participation of other cell types.

## Discussion

GOS is a mixture of oligosaccharides consisting of variable degrees of polymerization of galactose units (up to 7) linked to a glucose terminal (21). In our previous description of GOS allergy, we showed that basophil activation by GOS required a minimum of three sugar moieties (i.e. DP3 with 2 galactose and 1 glucose) (4). It remains a puzzle as to how these oligosaccharides of low molecular weight (~540 daltons) could cross-link IgE on basophils which by criteria requires at least two IgE binding epitopes in order to bind two molecules of cell surface IgE simultaneously. In this study, we examined possible mechanism(s) by which GOS as an allergen activates basophils.

Our first approach was to consider the possible role of lectins that may participate in GOS-specific IgE cross-linking on basophils. Basophils are shown to have surface expression of some C-type lectins such as CLECSF14, DEC205, Dectin-1, Dectin-2, and mannose receptor C 2 (MRC2) and I-type lectins, immunoglobulin-like lectin (Siglec)-8 (22, 23). However, none of these lectins are known to bind β-galactoside sugars. On the other hand, basophils also express galectins which are a family of animal lectins that bind β-galactoside containing sugars with high specificity. As many as 12 galectins have been found in humans (24). They are present as cytoplasmic or secreted forms. The cytoplasmic form is usually monomeric and the secretory form can become di- or pentameric. After their secretion, they can bind to O- or N-linked glycans of cell surface proteins, such as CD43, CD45, TCR, Tim-3, Fc epsilon receptor I (25, 26). Galectins-1,−3,−8, and−9 are commonly bounded to the surface molecules of immune cells. Furthermore, galectins may trigger receptor cross-linking due to their di- or multi-valent characteristics. Since GOS is composed of different galactosyl residues (21), it is likely that GOS binds to galectins. Our earlier studies showed that basophils could be activated by GOS in serum-free conditions, indicating that circulating plasma factors such as soluble galectins are not likely to participate in GOS-mediated basophil activation (4). It is, however, possible that membrane-bound galectins acts as carrier molecules for GOS to cross-link IgE. Galectin-3 in particular can undergo conformational change to form pentamers upon ligand binding. GOS may, therefore, become multivalent through binding to pentameric galectin-3.

In this study, we examined the involvement of galectins in GOS-mediated basophil activation in whole blood assay using five different galectin inhibitors. Our results, however, showed that three of the five high affinity galectin inhibitors TD139, GB1107, and G3-C12 are used, failed to inhibit GOS-triggered basophil activation (Figure 1B), hence, ruling out the involvement of galectins-1 and−3 in the GOS-mediated basophil activation.

Contrasting results were obtained with the remaining two galectin inhibitors, TDG and lactose. Both showed inhibitory effects on GOS-mediated basophil activation at low concentrations of GOS, but this inhibition was diminished/lost at higher GOS concentrations (Figure 1B). Since both TDG and lactose have lower affinity for galectins compared to the former three inhibitors, we postulated that these observed inhibitory effects on GOS-mediated basophil activation were not likely due to galectin inhibition *per se*.

In view of the structural similarities between GOS, lactose, and TDG, we explored the possibility that these galectin inhibitors inhibit the binding of GOS to GOS-specific IgE. Indeed, the GOS IgE ELISA inhibition showed that both lactose and TDG inhibited the GOS IgE binding in a dose-dependent manner, with inhibition up to 2–3 log concentrations higher for lactose and TDG than self-inhibition with GOS and enriched GOS fraction (DP4) (Figure 2).

We next examined the possibility that GOS may obtain di- or multi-valent IgE binding capacity through binding with other types of lectins on immune cell surface molecules. Basophil activation tests using GOS, with anti-IgE and Blo t controls, were performed using purified basophils. A reduction in basophil activation was observed with all three stimulants, with the highest reduction for GOS (two samples negative), followed by anti-IgE (1 sample negative), and Blo t showed the least reduction (Figure 3). The two subjects whose purified basophils showed negative BAT results to GOS also showed >50% reduction in the percentage of CD63^+^ cells (one subject negative) to control stimulant anti-IgE. We, therefore, speculate that the purification steps reduced the density of IgE molecules on basophils and may increase the threshold for basophil activation, hence, affecting the responses to anti-IgE and GOS stimulation. These effects were less marked with Blo t stimulation, and a plausible reason is that Blo t allergens are multivalent whereas anti-IgE is divalent. The number of IgE epitopes per GOS is still unknown but it is likely fewer than Blo t in view of its molecular weight. Furthermore, for BAT using whole blood samples, basophil activation was achieved with the concentrations of Blo t were 10–100 times less than GOS (Figure 1A) indicating that Blo t may have a higher affinity to IgE than GOS and hence the degree of basophil activation is less affected by this increased threshold. Taken together, despite the loss of basophil activation in two subjects, our data suggest that GOS is likely to be able to cross-link IgE on basophils independently.

Although the IgE binding epitopes of GOS allergy are still unresolved, our competitive ELISA assay indicates that disaccharides (lactose and TDG) can bind GOS-specific IgE. Of note, lactose and TDG are disaccharides composed of different sugar molecules suggesting that GOS-specific IgE is not monoclonal, with higher affinity for the structure with two galactose rings (TDG) than one galactose plus one glucose (lactose).

We, therefore, speculate that at least some oligosaccharides in the mixture of GOS are likely to possess two or more IgE epitopes. However, it is known that binding of two antibodies (such as IgE) to epitopes in close proximity can be blocked by steric hindrance, especially for small molecules (27). In this regard, Kaneko et al. have shown that a GOS linear tetrasaccharide with only β1-4 linkage is unable to activate basophils but the linear structure Galβ1-4Galβ1-4Galβ1-3Glc and branched structure Galβ(1-4)Galβ(1-6)-[Galβ(1-4)]-Glc (Table 1 in bold) are allergenic in GOS allergy subjects in Japan (28). For this latter structure, we have previously confirmed that it does not trigger the activation in basophils from Singapore GOS allergic subjects, suggesting that GOS allergy can be caused by different GOS structures in different regions (29). Although the exact allergenic GOS structures responsible for GOS allergy in Singapore are still unknown, our study also indicated that the enriched DP4 fraction with more branched GOS structures from specifically produced GOS is more potent than DP4 fraction from commercial vGOS in inducing basophil activation *in vitro* (6). Branched GOS structures may be more likely to overcome the steric hindrance and cross-link IgE on basophils.

In conclusion, we have demonstrated that GOS-mediated IgE cross-linking does not require galectin as carrier. GOS may be able to cross-link IgE independently.

## Data Availability Statement

The original contributions presented in the study are included in the article/Supplementary Material, further inquiries can be directed to the corresponding author.

## Ethics Statement

The studies involving human participants were reviewed and approved by Institutional Review Board of the National Healthcare Group, Singapore. Written informed consent to participate in this study was provided by participants/participants' legal guardian.

## Author Contributions

LL conducted experiments and performed data analysis. SL conducted experiments, performed data analysis, and drafted manuscript. HW conducted experiments. JS, WC, YZ, ET, and WL contributed to the sample collection, data interpretation, and revised the manuscript. DD provided the materials required for the study and contributed to the data interpretation and revised the manuscript. BL contributed to the study design, data interpretation, and revised the manuscript critically. C-HH conceived the study and study protocol and participated in manuscript writing. All authors contributed to the article and approved the submitted version.

## Funding

This work was supported by the National Medical Research Council, Singapore (NMRC/CIRG/1487/2018).

## Conflict of Interest

DD is employed by FrieslandCampina, Amersfoort, The Netherlands. The remaining authors declare that the research was conducted in the absence of any commercial or financial relationships that could be construed as a potential conflict of interest.

## Publisher's Note

All claims expressed in this article are solely those of the authors and do not necessarily represent those of their affiliated organizations, or those of the publisher, the editors and the reviewers. Any product that may be evaluated in this article, or claim that may be made by its manufacturer, is not guaranteed or endorsed by the publisher.
