# Supplementary material for: An Evaluation of the Mechanisms of Galacto-Oligosaccharide (GOS)-Induced IgE Cross-Linking on Basophils in GOS Allergy
Source: Front Allergy. 2022 Feb 28;3:840454. doi: 10.3389/falgy.2022.840454 (PMC8974727; doi:10.3389/falgy.2022.840454)
Supplement: Supplementary file 1 [file Data_Sheet_1.PDF]

Supplementary Material

1.1 Supplementary Figure

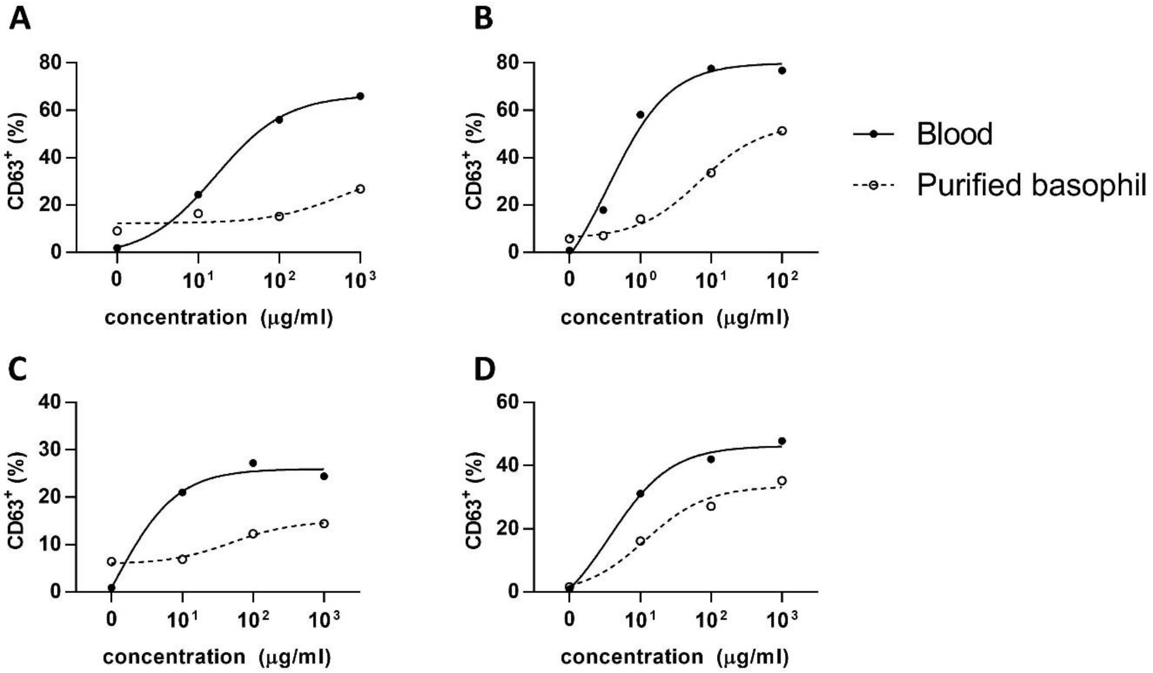

Supplementary Figure 1: Basophil activation assay to GOS performed using whole blood or purified basophils. Heparinized whole blood or purified basophils were stimulated with various concentration of GOS as indicated or without stimulation. The expression of CD63 on basophils were analysed by flow cytometry. Graph (A), (B), (C), and (D) represent subject S1, S2, S3 and S4, respectively.
